# Supplementary material for: Healthcare providers’ perceptions of the kidney transplant program at King Faisal Hospital Rwanda: A one-year evaluation
Source: PLOS Glob Public Health. 2025 Jul 28;5(7):e0004905. doi: 10.1371/journal.pgph.0004905 (PMC12303323; doi:10.1371/journal.pgph.0004905)
Supplement: Attachment — Submitted filename: HCP perspectives on KT - Response to Reviewers.pdf [file pgph.0004905.s003.pdf]

Section	Reviewer	Feedback	Response
Supporting Information	Journal Requirements	Missing list of legends in the manuscript for Supporting Information files.	We agree with the utility of a list of legends for the Supporting Information and have added this after the References section of the manuscript. Only one Supporting Information file was included, as we felt the additional survey previously included was redundant. The Supporting Information file was renamed to reflect journal requirements.
References	Journal Requirements	Cited papers that have been retracted.	We reviewed each citation in our reference list to ensure that none have been retracted. The reference list has been updated accordingly (One reference was added to support the discussion section)
Methods	1	It is unclear if the study started with interviews and the results influenced the development of a quantitative tool, as it should be in an explanatory mixed method design.	The study started with the initial survey that quantitatively gathered descriptive information about the role and general overview of the participants' experience towards the transplant program, and the results from the initial survey influenced the development of the semi-structured interviews. We have indicated this in the methodology section
Results	1	The way the results are presented do not reflect an exploratory design, and instead show the data were collected concurrently and results were triangulated to meet set objectives.	We have restructured the results to reflect the exploratory study design employed in this study. This program is new in this setting (King Faisal Hospital), and no previous studies have been made to understand how the HCPs perceived this program, their role, and challenges. We, therefore, with no pre-set hypotheses, have used an exploratory study to generate new knowledge, ideas, and highlight their experience, which can be referred to for further studies.
Methods	1	Exclusion criteria need to be explained and ethical considerations on whether participation was voluntary or not.	We excluded from the study any individual who was a patient at KFH and any individual who had not yet participated in the transplant program. These criteria were added to the Methods section, as well as a statement that participation was voluntary. Voluntary participation was also addressed in the ethics statement.
Abbreviations	2	Some abbreviations need to be written in full before being used in the next sentence.	We edited the manuscript to write in full "kidney transplant" before being used in the Introduction and "arteriovenous fistula" in the Discussion. We also edited the abbreviations section to remove abbreviations that were not used in the manuscript ("ICU", "OPD", "OT").
Abstract	2	Redundancy in the background section "needs for further developing the developing kidney transplant program."	We removed the second "developing" in this sentence.
Discussion	2	Consider reorganizing parts of the discussion to group similar points together and reduce some repetition, particularly regarding challenges with dialysis services.	We have reorganized the discussion to first discuss all findings related to patient care coordination, especially dialysis services, followed by all findings related to education and training.
Results	2	It is mentioned that 20 of 50 respondents were nurses. However, a full breakdown of the professions is missing. A simple descriptive text would address this issue.	We added a Table 1 with a full breakdown of professions represented in the survey respondents.
Throughout	2	Small language and grammar corrections	We have further edited the manuscript for correct grammar and language.
Limitations	2	Address the professional distribution imbalance. For example, administrative staff are fewer (2/50), so their responses could be lost among the clinicians' voices.	We edited the Limitation section to address the imbalance in professions represented in the study and provided an example. However, if compared to other professional categories, administration had a relatively low proportion of the population.
