# Supplementary material for: Healthcare providers’ perceptions of the kidney transplant program at King Faisal Hospital Rwanda: A one-year evaluation
Source: PLOS Glob Public Health. 2025 Jul 28;5(7):e0004905. doi: 10.1371/journal.pgph.0004905 (PMC12303323; doi:10.1371/journal.pgph.0004905)
Supplement: Attachment — Submitted filename: HCP perspectives on KT - Response.pdf [file pgph.0004905.s004.pdf]

Section	Reviewer	Feedback	Response
Abstract	1	"Methods: This study employed an exploratory design, with a mixed-methods approach" this can be confusing (easily misunderstood to speak to the mixed methods) and can be clarified- "This study employed a sequential explanatory mixed methods approach embedded within an overarching exploratory research design." (if you want to keep the concept of an exploratory study)	Terminology was changed in Abstract (lines 45-46) to better reflect the sequence of data collection, which involved an initial survey followed by interviews.<br><br>We have clarified that "This study employed a sequential explanatory mixed methods approach embedded within an overarching exploratory research design"
Methods	1	Line 85-87 (also confusing) "The study was exploratory research by design. Data was collected from an initial survey and 87 semi-structured interviews" the sequence suggests that this is the definition of exploratory. Please clarify that this was a sequential explanatory mixed methods approach.	Terminology was changed in the Methods (line 88) to better reflect the sequence of data collection, which involved an initial survey followed by interviews.<br><br>We have clarified that "The study utilized a sequential, explanatory mixed methods approach"
